# Low Thermal Conductivity in Single Crystalline Mg_3_Bi_2_ and Its Thermopower Enhanced by Electron‐Phonon Interaction

**DOI:** 10.1002/advs.202416518

**Published:** 2025-05-05

**Authors:** Qiang Feng, Jiayi He, Wenyang Wang, Huili Liu

**Affiliations:** ^1^ School of Physical Science and Technology ShanghaiTech University Shanghai 201210 China

**Keywords:** electron‐phonon interaction, single crystalline Mg_3_Bi_2_, thermal conductivity, thermoelectrics, thermopower

## Abstract

Promising thermoelectric materials are usually those of “phonon‐glass electron‐crystal” (PGEC) compounds, with low thermal conductivity and high carrier mobility. In metallic materials, strong electron‐phonon interaction usually causes an increase of Seebeck coefficient (*S*) at low temperature, due to an extra electrical current driven by heat‐carrying phonons, named phonon drag effect. Here, this study reports that single crystalline metallic Mg_3_Bi_2_ has low lattice thermal conductivity of ≈0.49 W m^−1^ K^−1^ at 285 K, and corresponding mean free path of phonons (*L*_ph_) is ≈0.48 nm, with carrier mobility of ≈54.2 cm^2^ V^−1^ s^−1^ around room temperature. It is found that *S* exhibits an increase as a “hump” ≈20 K, and phonon drag effect (*S*_ph_) contributes to ≈80%, significantly higher than diffusive electrons. Meanwhile, *S*_ph_ is positively proportional to *L*_ph_, where coefficient of *S*_ph_/*L*_ph_ is ≈4.6 × 10^2^ µV K^−1^ µm^−1^, twice that of CrSb_2_ and FeSb_2_, and relative strength of electron‐phonon interaction is ≈0.12. The *L*_ph_‐intercept of *S*_ph_/*L*_ph_ approaches to ≈4.68 nm, where phonons can be strongly scattered before interacting with electrons, leading to a negligible phonon drag effect. The findings shed light on fundamental understanding of thermoelectric transport and exploring novel thermoelectric materials.

## Graphical abstract

In this work, low lattice thermal conductivity and high carrier mobility are revealed in single crystalline Mg_3_Bi_2_, a promising thermoelectric material. An increase of thermopower as a “hump” ≈20 K is found, where phonon drag effect contributes large portion by replacing diffusive electrons. A high coefficient of phonons driving thermopower is discovered due to its strong electron‐phonon interaction.

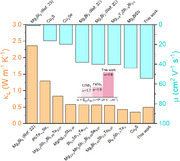

## 1 Introduction

Thermoelectric materials can convert heat into electricity with electrons or holes as working fluids, and vice versa.^[^ ^1^, ^2^ ^]^ Thermoelectric technology, possessing high stability, no pollution, no noise, precise temperature control, and fast response speed, provides a sustainable strategy to resolve emerging energy issues.^[^ ^3^ ^]^ The efficiency of thermoelectric materials is governed by a dimensionless figure of merit, *zT = S^2^σT/*(*κ_L_* +*κ_e_*), where *S* is Seebeck coefficient, *σ* is electrical conductivity, *κ_L_* is lattice thermal conductivity, *κ_e_* is electrical thermal conductivity, and *T* is absolute temperature. For decades, thermoelectric figure of merit has been improved to *zT* >1.5 in some promising materials with several new strategies or concepts, such as Bi_2_Te_3_,^[^ ^4^, ^5^, ^6^ ^]^ MgAgSb,^[^ ^7^, ^8^ ^]^ PbTe,^[^ ^9^ ^]^ Cu_2_X (X = S, Se)^[^ ^10^, ^11^ ^]^ and recent n‐type Mg_3_(Bi, Sb)_2_ alloys.^[^ ^12^, ^13^, ^14^, ^15^, ^16^ ^]^

Mg_3_Bi_2_ compound, a Zintl phase with the layered structure, is composed of cationic Mg^2+^ and anionic (Mg_2_Bi_2_)^2‐^ layers, and quite stable under air enviroment.^[^ ^17^ ^]^ Complex structures and heavy element mass in compounds typically reduce *κ_L_* of materials. However, with simple lattice structure and light element mass, Mg_3_Bi_2_ has an anomalous low *κ_L_*, probably due to the softening of anharmonic lattice vibrations or high density of defects.^[^ ^18^, ^19^, ^20^ ^]^ Recently, thermoelectric properties of polycrystalline Mg_3_Bi_2_ at medium or high temperature, and thermal conductivity of single crystalline Mg_3_Bi_2_ have been reported.^[^ ^15^, ^19^, ^20^, ^21^ ^]^ It was observed that Seebeck coefficient is significantly enhanced at low temperature for single crystalline Mg_3_Bi_2_, which was synthesized by Bi flux or Mg flux.^[^ ^22^, ^23^ ^]^ This phenomenon of *S* enhancement at low temperature could be caused by structural phase transitions,^[^ ^24^ ^]^ magnetic phase transitions,^[^ ^25^ ^]^ or strong electron‐phonon interactions.^[^ ^26^, ^27^, ^28^ ^]^ However, it still lacks fundamental and physical understanding of thermoelectric transport at low temperature in single crystalline Mg_3_Bi_2_.

Typically, phonon drag effect could be augmented in high‐purity single crystalline semiconductors at cryogenic temperature,^[^ ^28^, ^29^, ^30^ ^]^ because of strong electron‐phonon interaction, and cause electrons redistribution across a temperature gradient, which would contribute additional thermopower in materials. The phonon drag contribution of Seebeck coefficient, *S*_ph_, could be simply estimated by Herring's model,^[^ ^31^ ^]^ *S*_ph_∝*βυL*_ph_ *μ* ^−1^ *T* ^−1^, where *β* is a parameter of 0–1 indicating strength of electron‐phonon interaction, *υ* is group velocity of phonons, *L*_ph_ is mean free path of phonons, and *μ* is Hall mobility of carriers. Therefore, due to a long mean free path of phonons with negligible phonon‐phonon or phonon‐impurity interaction, phonon‐drag effect would drive a large increase of thermopower at low temperature.

In this work, we successfully synthesized single crystalline Mg_3_Bi_2_, under several different conditions using Bi‐flux method. We carried out the measurement of thermal and thermoelectric properties at low temperature, and observed a clearly increase of *S* in our samples ≈20 K. At that temperature, single crystalline Mg_3_Bi_2_ has its highest *κ_L_*, in the intersection of other phonons and boundaries dominating phonon scattering for thermal conduction. The lowest *κ_L_* is ≈0.49 W m^−1^ K^−1^ for single crystalline Mg_3_Bi_2_ at 285 K, with highest carrier mobility of 54.2 cm^2^ V^−1^ s^−1^ around room temperature, and the corresponding *L*_ph_ is estimated to ≈0.48 nm, approaching to the minimum of atomic distance in crystal structure. It is elucidated that strong electron‐phonon interaction causes the phonon drag effect, driving the increase of Seebeck coefficient at low temperature, contributing to ≈80% of *S*, and the relative strength of electron‐phonon interaction is estimated as ≈0.12 in Mg_3_Bi_2_. The estimated additional *S* by phonon drag effect is positively proportional to the mean free path of phonons, and the coefficient (*α*) of *S*_ph_ */L*_ph_ is ≈4.6 × 10^2^ µV K^−1^ µm^−1^ in single crystalline Mg_3_Bi_2_, more than twice that of CrSb_2_, FeSb_2_, et al.^[^ ^27^, ^28^ ^]^ The *L*_ph_‐intercept of the linear fit to *S*_ph_ approaches to ≈4.68 nm, where strong phonon‐boundary or phonon‐impurity interaction cause that phonon‐drag effect could not be detected.

## 2 Results and Discussion

**Figure 1:**
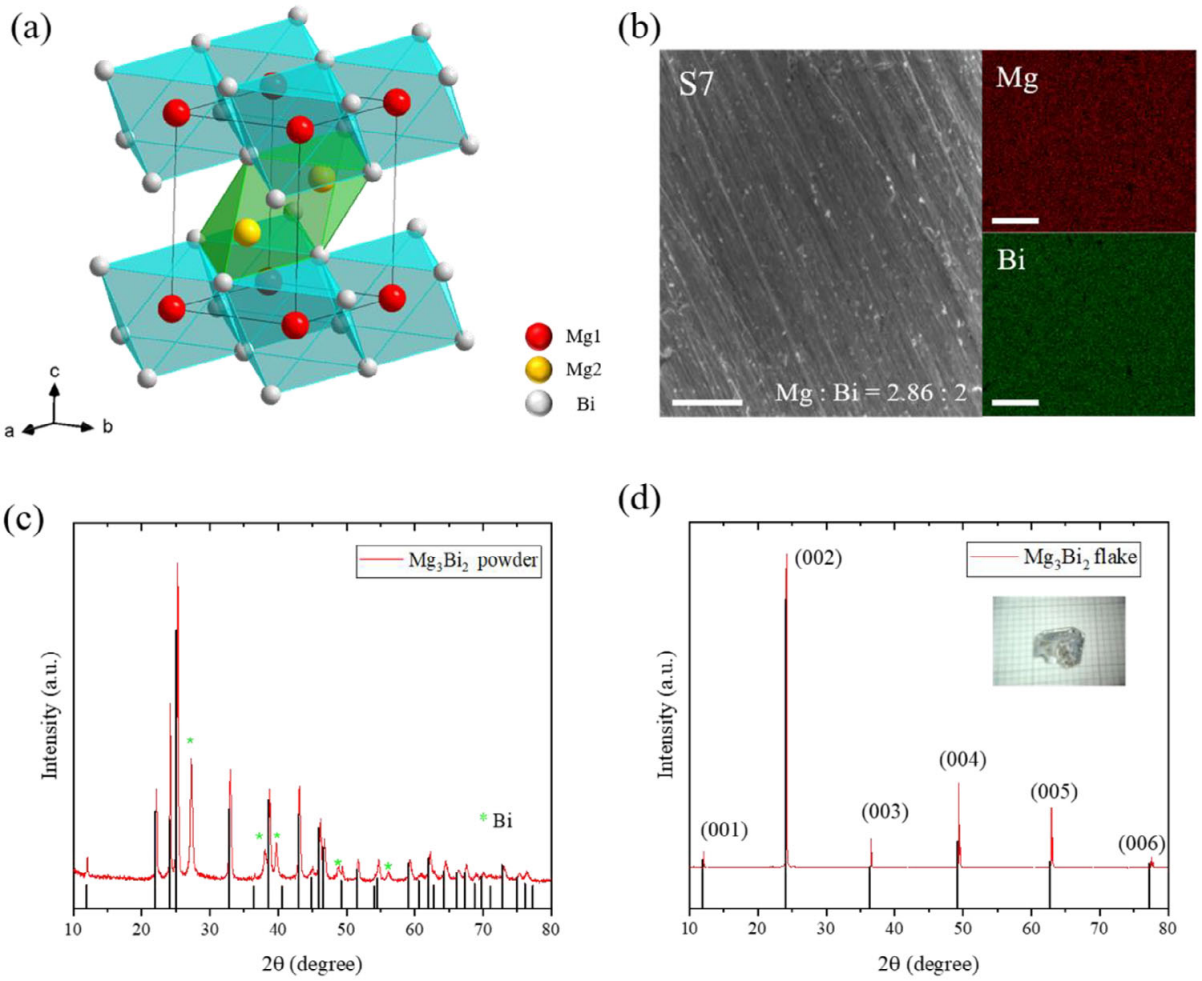
Crystal structure and SEM image of Mg_3_Bi_2_. a) Crystal structure of Mg_3_Bi_2_, where red, yellow and white balls indicate Mg1, Mg2, and Bi atoms, respectively. b) Images of SEM and EDS for sample S7; The scale bar is 50 µm. c) X‐ray diffraction patterns of polycrystalline Mg_3_Bi_2_; Dark lines are from PDF#65‐8732, and green stars indicate the remain of excess Bi using flux method. d) X‐ray diffraction patterns of Mg_3_Bi_2_ flake; Inset is an optical image of the flake with a lateral size of ≈6–8 mm.

**Figure** **1**a shows crystal structure of [Mg_2_Bi_2_]^2−^, a typical CaAl_2_Si_2_‐type compounds family, where a cationic Mg^2+^ sheet and an anionic [Mg_2_Bi_2_]^2−^ layer stacked alternately along c‐axis. Mg1 and Mg2 are on octahedral and tetrahedral sites, respectively. The scanning electron microscope (SEM) image in Figure 1b shows a clean surface of the flake, and elemental mapping of energy‐dispersive X‐ray spectroscopy (EDS) indicates a uniform distribution of Mg and Bi elements with atomic ratio of Mg: Bi ≈2.86:2 for S7. The details of compositions for all samples are listed in Table 1. The compositions were also determined by inductively coupled plasma optical emission spectrometry (ICP‐OES) analysis with Bi_2_ calibration (see Table SI, Supporting Information). Powder diffraction patterns of samples is shown in Figure 1c, where all diffraction peaks are indexed by anti‐α‐La_2_O_3_‐type crystal structure with space group P‐3m1 (No. 164), consistent with previous report.^[^ ^32^, ^33^ ^]^ It is shown that pure bismuth phase remains, probably originating from Bi source during the centrifuging process by flux method. Diffraction patterns on the surface of Mg_3_Bi_2_ flake, with a lateral size of ≈6–8 mm (see inset in Figure 1d), are shown in Figure 1d, in which only (00l) planes are indexed, indicating a high quality of single crystals for the samples.

**Table 1:** Summary of electrical and thermoelectric properties around room temperature. In‐plane resistivity (*ρ*), concentration (*p*) and Hall mobility (*μ*) of carriers, Seebeck coefficient (*S*), thermal conductivity (*κ*), and lattice thermal conductivity (*κ_L_*) for the samples in this work, and results from literatures as comparison, as well as nominal composition and actual composition by EDS.

Sample^a^ ^)^	*ρ* [10^−6^Ohm m]	*p* [10^20^cm^−3^]	*μ* [cm^2^ V^−1^ s^−1^]	*S* [µV K^−1^]	*κ* [W m^−1^ K^−1^]	*κ_L_* [W m^−1^ K^−1^]	Nominal composition	Actual composition [EDS]
S1	6.93	3.161	28.5	50.64	2.09	1.24	Mg_18_Bi_82_	Mg_2.84_Bi_2_
S2	6.53	2.410	39.7	—	—	—	Mg_18_Bi_82_	Mg_2.89_Bi_2_
S3	—	—	—	44.96	1.90	0.90		Mg_2.89_Bi_2_
S4	6.06	1.911	54.2	51.96	2.14	1.08	Mg_1_Bi_3_	Mg_2.80_Bi_2_
S5	9.62	1.885	34.4	35.60	2.42	1.59	Mg_1_Bi_2_	Mg_2.82_Bi_2_
S6	—	—	—	44.81	1.58^b^ ^)^	0.49^b^ ^)^	Mg_1_Bi_3_	Mg_2.80_Bi_2_
S7	8.65	1.987	36.4	53.79	1.89	1.15	Mg_1_Bi_2_	Mg_2.86_Bi_2_
S8	6.64	2.865	31.6	40.43	1.94	0.94	Mg_1_Bi_4_	Mg_2.78_Bi_2_
S9	7.65	2.818	30.6	41.16	1.73	0.88	Mg_3_Bi_7_	Mg_2.70_Bi_2_
S10	6.51	2.518	38.1	39.19	1.81	0.79	Mg_1_Bi_3_	Mg_2.76_Bi_2_
Xin, et al.^[^ ^22^ ^]^	6.13	2.521	40.1	54.47	3.59	2.36	Mg_3_Bi_7_	—
Liu, et al.^[^ ^34^ ^]^	17.8	0.121	290.0	−226.97	0.99	—	Mg_3.2_Bi_1.5_Sb_0.498_Te_0.002_Cu_0.01_	—
Ponnambalam, et al.^[^ ^35^ ^]^	151	0.084	44.0	195.42	0.81^c^ ^)^	—	Mg_3_BiSb	—

^a)^ Note: S2 & S3 from the same batch; S4 & S6 from the same batch.

^b)^ Data collected at 285 K;

^c)^ Data collected at 320 K.

**Figure 2:**
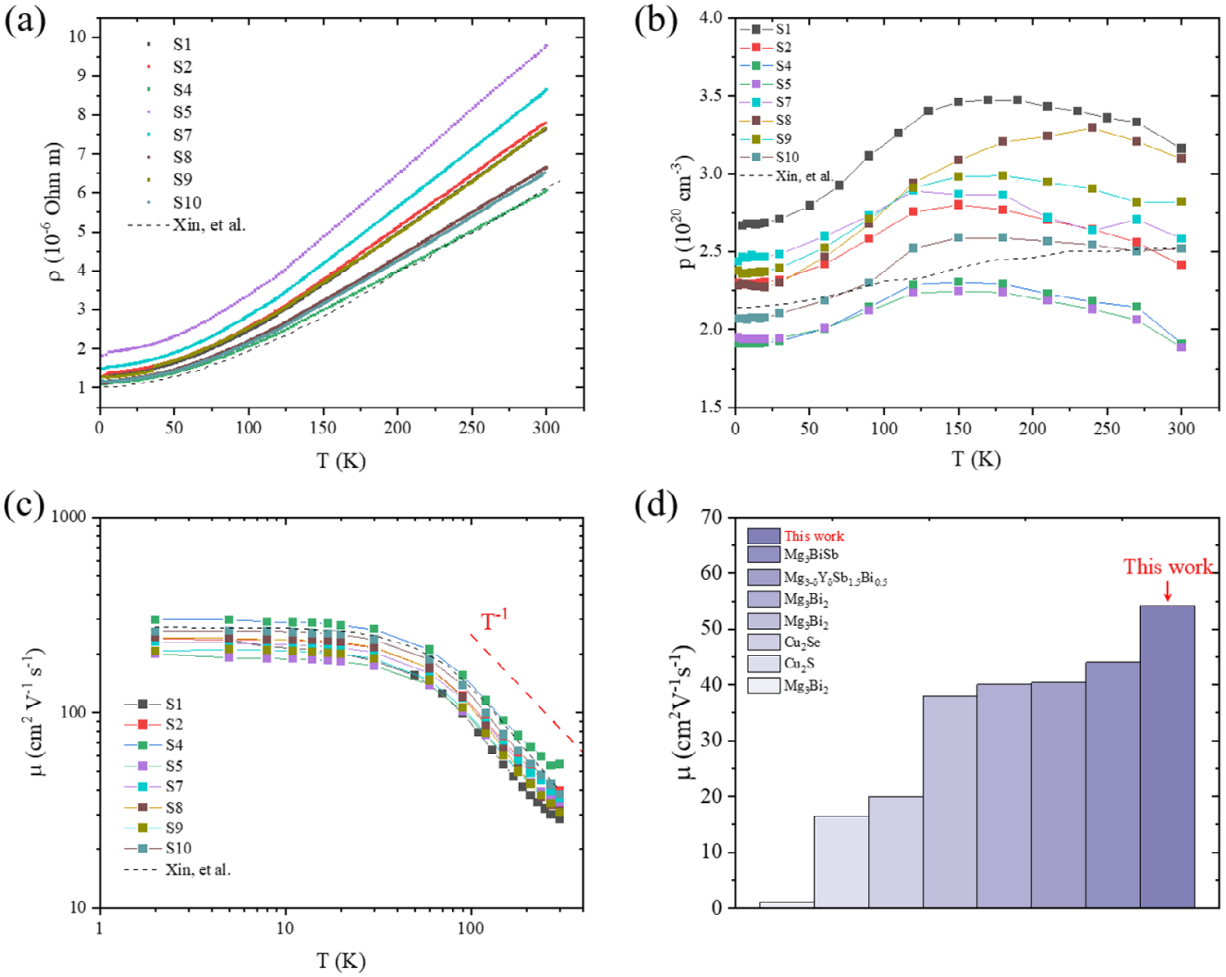
In‐plane electrical properties in single crystalline Mg_3_Bi_2_. Temperature dependence of a) resistivity, b) carrier concentration, and c) Hall mobility. d) Comparison of carrier mobility around room temperature for several compounds, Mg_3_BiSb,^[^ ^35^ ^]^ Mg_3‐δ_Y_δ_Sb_1.5_Bi_0.5_,^[^ ^38^ ^]^ Mg_3_Bi_2_,^[^ ^22^, ^32^, ^33^ ^]^ Cu_2_Se,^[^ ^10^ ^]^ Cu_2_S,^[^ ^11^ ^]^ and this work. Red dash line presents the relationship of *μ∼T* ^−1^ with polar‐optical phonons dominating electrons scattering above 100 K. Dark dash line from ref. [22]. Lines connecting data are guide to the eye.

The in‐plane resistivity of single crystalline Mg_3_Bi_2_ samples from several batches are shown in **Figure** **2**a. The resistivity increases with elevated temperature at the range of 2–300 K, showing a metallic behavior of electrical transport with a residual resistivity ratio (RRR)^[^ ^36^ ^]^ of ≈5. The resistivity of the samples varies, probably due to differences of crystalline quality caused by defects or impurity levels. Carrier concentrations of the samples were determined from Hall measurement based on a typical Hall‐bar geometry (see inset in Figure S1a, Supporting Information), and a positive Hall coefficient indicates holes are dominating carriers in the samples (see Figure S1, Supporting Information, for the samples at low temperature). Those crystals were synthesized by changing the molar ratio of Mg and Bi, which could not directly change the dominating electrical transport type of carriers, consistent with the results reported by Pan et al.^[^ ^23^ ^]^ using the Mg‐flux method. The estimated carrier concentrations (*p*) are 1.88–3.16 × 10^20^ cm^−3^ at room temperature (see Table 1), and nearly invariable for different batches (shown in Figure 2b), which are consistent with previous reports,^[^ ^22^, ^32^ ^]^ and higher than that of ≈10^19^ cm^−3[^ ^16^, ^37^, ^38^, ^39^, ^40^, ^41^ ^]^ in doped polycrystalline samples. In Figure 2c, Hall mobility (*μ*) of carriers is 28.5–54.2 cm^2^ V^−1^ s^−1^ at room temperature, and has a temperature dependence of *T* ^−1^ at the range of 100–300 K, indicating that diffusive electrons would be strongly scattered by phonons, probably polar‐optical phonons.^[^ ^42^ ^]^ The comparison of the Hall mobility of carriers is shown in Figure 2d, *μ* of this work is much higher than that of single crystals synthesized using Bridgman method,^[^ ^33^ ^]^ probably due to differences in sample quality and density of defects.

**Figure 3:**
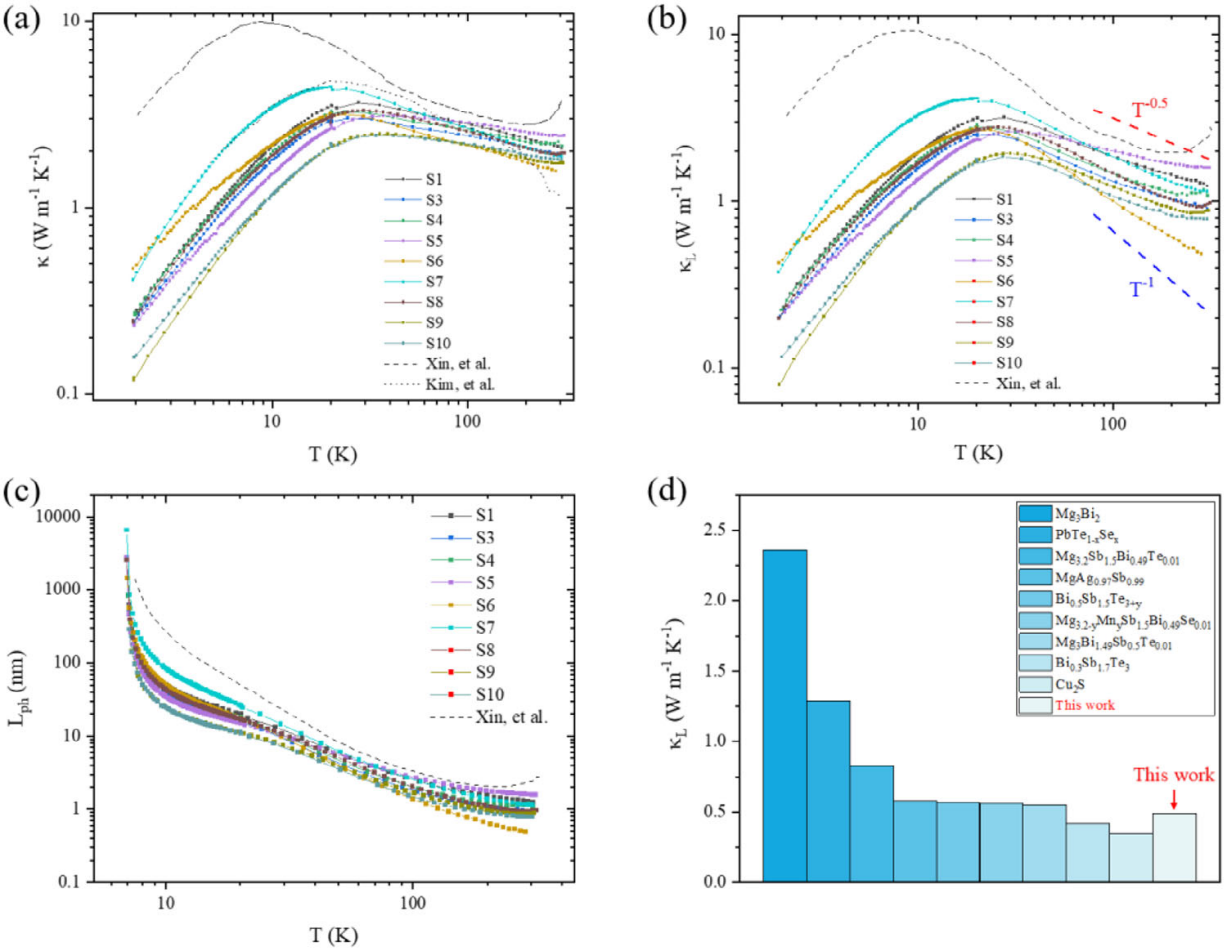
In‐plane thermal properties in single crystalline Mg_3_Bi_2_. a) Thermal conductivity, b) lattice thermal conductivity, and mean free path of phonons c) as a function of temperature. Black dots and dash lines are adapted from references. Red and blue dash lines indicate relationship of *κ_L_* ∼*T* ^−0.5^ and *κ_L_* ∼ *T^−^* ^1^, respectively. Lines connecting data are guide to the eye. d) Comparision of *κ_L_* around room temperature for some thermoelectric materials, Mg_3_Bi_2,_^[^ ^22^ ^]^ PbTe_1‐x_Se_x,_^[^ ^9^ ^]^ Mg_3.2_Sb_1.5_Bi_0.49_Te_0.01,_^[^ ^12^ ^]^ MgAg_0.97_Sb_0.99,_^[^ ^7^ ^]^ Bi_0.5_Sb_1.5_Te_3+y,_^[^ ^4^ ^]^ Mg_3.2+y_Mn_y_Sb_1.5_Bi_0.49_Se_0.01,_^[^ ^16^ ^]^ Mg_3_Bi_1.49_Sb_0.5_Te_0.01,_^[^ ^14^ ^]^ Bi_0.3_Sb_1.7_Te_3,_^[^ ^5^ ^]^ Cu_2_S_,_^[^ ^11^ ^]^ and this work.

**Figure** **3**a shows temperature dependence of *κ* in single crystalline Mg_3_Bi_2_. We take Lorenz number *L*_0_, estimated by an equation: $L_{0}=\left(1.5+exp[-\frac{\left|S\right|}{116}]\right)\;$ × 10^−8^ W Ω K^−2^, to calculate *κ*_e_, based on the experimental Seebeck coefficient.^[^ ^43^ ^]^ It is shown that *κ*_e_ contributes to a large portion of *κ* at room temperature, whereas could be negligible at low temperature (see Figure S2, Supporting Information). Figure 3b shows *κ_L_* of samples as a function of temperature, which is slightly higher than that of polycrystalline compounds,^[^ ^14^, ^41^, ^44^ ^]^ but lower than that of single crystals in previous reports.^[^ ^19^, ^22^, ^23^ ^]^ In those samples, a higher density of defects or impurities might hinder heat‐carrying lattice vibration, resulting in a reduction of *κ_L_*, and *κ_L_* is ≈0.49‐1.59 W m^−1^ K^−1^ around room temperature. Above 100 K, temperature dependence of *κ_L_* for S6 is close to the relationship of *T* ^−1^, where heat conduction is mainly dominated by phonon‐phonon scattering (Umklapp process). At 285 K, S6 has an extremely low lattice thermal conductivity with *κ_L_* ≈0.49 W m^−1^ K^−1^, much lower than that of ≈2.36 W m^−1^ K^−1^ at 300 K reported by Xin et al.^[^ ^22^ ^]^ This makes *κ_L_* of single crystalline Mg_3_Bi_2_ in this work reach to the lowest one in solid‐state compounds, compared to several promising thermoelectric materials, such as Bi_2_Te_3_,^[^ ^4^, ^6^ ^]^ MgAgSb,^[^ ^7^ ^]^ PbTe,^[^ ^9^ ^]^ Cu_2_S^[^ ^11^ ^]^ et al., as shown in Figure 3d. However, for other samples, it is shown that *κ_L_* approaches to the relationship of *T* ^−0.5^, deviating from the Umklapp process, probably due to a high density of defects,^[^ ^45^, ^46^ ^]^ similar with that of polycrystalline FeSb_2_.^[^ ^47^ ^]^ Meanwhile, *κ_L_* varies for those samples because of different density levels of Mg vacancies,^[^ ^45^ ^]^ caused by the loss of Mg element during synthesis process^[^ ^12^ ^]^ with vapor pressure and chemical activity, which is consistent with the results in previous experiments or theory.^[^ ^12^, ^13^, ^48^ ^]^ Therefore, the *L*_ph_ could be regulated by the density of Mg vacancy defects, determined by the conditions of synthesis. The estimated *L*_ph_ is shown in Figure 3c, according to the kinetic theory of phonons gas model, *κ_L_* = *CυL*_ph_/3, where *C* is specific heat capacity. It is shown that *L*_ph_ is less than ≈2 nm at room temperature, and approaching to ≈6.55 µm ≈7 K, lower than that in report.^[^ ^22^ ^]^ Particularly, the *L*_ph_ for S6 is estimated as ≈0.49 nm at 285 K, approaching to the minimum atomic distance in crystal lattice of Mg_3_Bi_2_, therefore makes it reaches to the minimum *κ_L_* in crystalline solid. It is believed that in‐plane thermal conduction for S6, with the highest carrier mobility of ≈54.2 cm^2^ V^−1^ s^−1^ around room temperature, is strongly limited by others phonons combining with defects.

**Figure 4:**
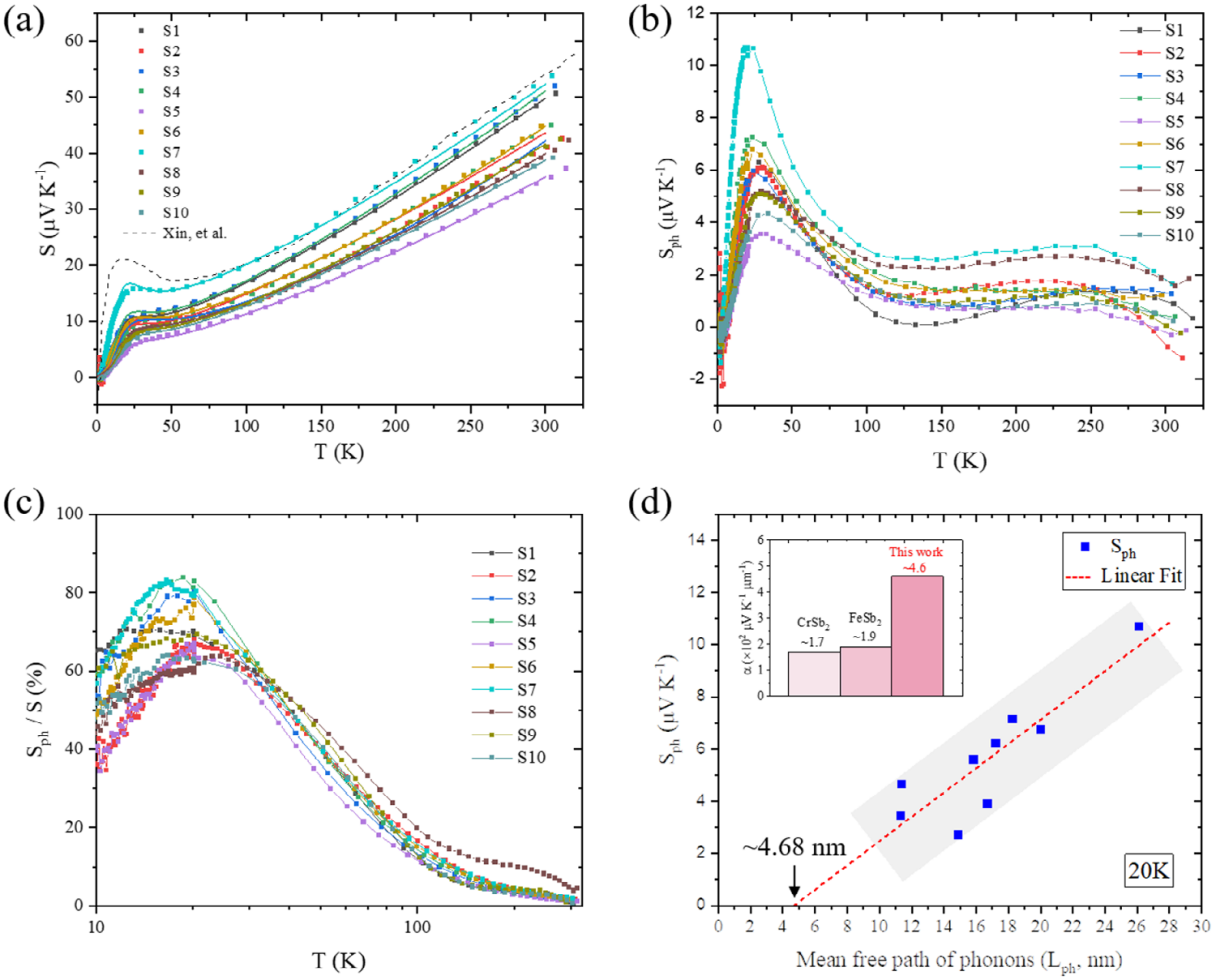
In‐plane thermoelectric properties in single crystalline Mg_3_Bi_2_. Temperature dependence of a) Seebeck coefficient, *S*, b) phonon drag Seebeck coefficient, *S*_ph_, and c) percentage of *S*_ph_/*S*; Solid lines are curves of the fit to *S* in (a). (d) *S*_ph_ as a function of mean free path of phonons at 20 K, where *α* = ≈4.6 × 10^2^ µV K^−1^ µm^−1^, and the *L*_ph_‐intercept of the fit is ≈4.68 nm; Inset is the comparison of *α* in CrSb_2_,^[^ ^27^ ^]^ FeSb_2_,^[^ ^28^ ^]^ and this work. Lines connecting data are guide to the eye in (b,c).

**Figure** **4**a shows *S* as a function of temperature. It is observed that *S* is enhanced significantly ≈20 K, and linearly increase with increasing temperature above 100 K. The “hump” of *S* are located at the temperature where *κ_L_* has its maximum value, which has been observed in other materials, such as FeGa_3_,^[^ ^29^ ^]^ FeSb_2_,^[^ ^47^, ^49^ ^]^ and CrSb_2_.^[^ ^27^ ^]^ It is reported that the increase of *S* is caused by the phonon drag effect,^[^ ^50^ ^]^ and the largest enhancement of *S* is reported in a narrow bandgap semiconductor FeSb_2_. Here, *S* as a function of temperature can be analyzed using a simple model proposed by Matoba et al.,^[^ ^51^, ^52^ ^]^

(1) $$S\;\left(T\right)=S_{d}+S_{ph}+S_{i}.$$

where *S_d_*, *S*_ph_ and *S_i_* are Seebeck coefficient contributed by diffusive electrons, phonon drag effect, and impurity effect, respectively. The detailed expression of the components can be described as Equations (2)–(4),

(2) $$S_{d}=AT+BT^{\gamma }$$

(3) $$S_{ph}=\frac{C{\left(T/\theta_{D}\right)}^{3}}{D+{\left(T/\theta_{D}\right)}^{4}}$$

(4) $$S_{i}=ET^{1/2}$$

where A, B, C, D, E are the fitting parameters, *γ* ∼2 for metallic materials,^[^ ^51^ ^]^ and *θ_D_* is Debye temperature. In this work, the experimental *S* were fitted using Equations (1)–(4) with *θ_D_* ≈175 K from literature.^[^ ^22^ ^]^ The fitting curves are shown in Figure 4a as solid lines, and details of the fitting parameters are listed in Table SII (Supporting Information).

Figure 4b shows *S*_ph_ as a function of temperature, by subtracting the components of diffusive electrons and impurity effect from experimental *S*, *S*–*S_d_*‐*S_i_*. It is obviously shown that phonon drag effect drives large increase of *S* as a significant “hump” ≈20 K. *S*_ph_ is positively proportional to the mean free path of phonons, as depicted in Figure 4c. In those samples, we observed that maximum *S*_ph_ is ≈10.6 µV K^−1^ for S7, which also has a highest *L*_ph_ of phonons ≈20 K. The mean free path of phonons indeed govern *S*_ph_, according to Herring's theory,^[^ ^31^ ^]^ *S*_ph_ ∼*βυL*_ph_ *μ* ^−1^ *T* ^−1^, similar with phonomenon observed in FeSb_2_^[^ ^28^ ^]^ and CrSb_2_.^[^ ^27^ ^]^ Additionally, we estimated the ratio of *S*_ph_/*S*, as shown in Figure 4c. It is found that *S*_ph_/*S* is up to ≈80% ≈20 K, but less than 20% above 100 K. It is noted that *S* in this work is lower than that reported by Xin et al.,^[^ ^22^ ^]^ which could be attributed to a shorter mean free path of phonons (see Figure 3d). In Figure 4d, it is shown that *S*_ph_ is almost positively proportional to *L*_ph_, and *α* is ≈4.6 × 10^2^ µV K^−1^ µm^−1^, much larger than that in FeSb_2_ (≈1.9 × 10^2^ µV K^−1^ µm^−1^) and CrSb_2_ (≈1.7 × 10^2^ µV K^−1^ µm^−1^)^[^ ^27^, ^28^ ^]^ (see inset in Figure 4d). It is noted that the *α* euquals *βυμ* ^−1^ *T* ^−1^, and quantified the variation of Seebeck coefficient attributed to phonon‐drag effect, with respect to the mean free path of phonons at certain temperature, which gives a high sensitivity of the comprehensive ability of phonon current driving charge carriers in the transport. This indicates a relatively strong electron‐phonon interaction, and *β* is calculated to ≈0.12 for single crystalline Mg_3_Bi_2_, larger than that of FeSb_2_ (≈0.05).^[^ ^28^ ^]^ In Herring’ theory, *β* is taken as 1 for only phonons scattering electrons. In Mg_3_Bi_2_, additional impurity scattering process has been taken into account for the electron‐phonon interaction. The defects, especially Mg vacancies, would effectivity scatter heat‐carrying phonons, leading to a reduction of mean free path of phonons in Mg_3_Bi_2_, and weaken phonon drag effect with high density of vacancies.^[^ ^49^ ^]^ Strong anharmonicity of lattice and weak interlayer bonds in Mg_3_Bi_2_^[^ ^18^, ^19^ ^]^ gives a low *κ_L_* with a short *L*_ph_. The maximum *L*_ph_ of the samples is estimated as ≈26 nm ≈20 K, much lower than that of FeSb_2_ (≈290 µm)^[^ ^28^ ^]^ and CrSb_2_ (≈630 µm).^[^ ^27^ ^]^ Therefore, it is observed that *S*_ph_ is a quite low in this work, <≈11 µV K^−1^ for single crystalline Mg_3_Bi_2_. The *L*_ph_‐intercept of the linear fit to *S*_ph_ is estimated as ≈4.68 nm, consistent with that of the *L*_ph_ above 100 K in Figure 3c, where *S*_ph_ is trivial, as shown in Figure 4b, and phonon drag effect could be negligible.

In a Fermi liquid system of the metallic materials,^[^ ^53^ ^]^ Fermi velocity (*υ_F_*) of electrons can be calculated by $\frac{\hbar }{m_{e}}{\left(3\pi^{2}p\right)}^{1/3}$, where ℏ and *m_e_* is reduced Planck constant and mass of electrons, respectively. In this work, for single crystalline Mg_3_Bi_2_, the concentration of carriers is at the range of 1.88–3.16 × 10^20^ cm^−3^, therefore Fermi velocity of electrons can be expected to 2.05–2.44 × 10^5^ m s^−1^. Basically, average scattering time (*τ*) of diffusive electrons can be calculated from Drude model, *τ = m_e_*/*ρe* ^2^ *p*, where room temperature resistivity and carriers concentration give the minimum scattering time of electrons as *τ_min_* = 1.62–3.07 × 10^−14^ s. Therefore, the minimum mean free path of diffusive electrons, *L_e_*_,_ *_min_*, can be predicted by *υ_F_τ_min_*, and *L_e_*_,_ *_min_* is at range of 3.88–6.34 nm. It is believed that the mean free path of phonons, approaching to the *L*_ph_‐intercept of ≈4.68 nm (see Figure 4d) or *L*_ph_ above 100 K (see Figure 3c), is less than *L_e_*_,_ *_min_*, and phonons would be mostly scattered by boundaries or defects before interacting with electrons. As a result, diffusive electrons would be dominating contributions to thermoelectric transport at low temperature in single crystalline Mg_3_Bi_2_.

## 3 Conclusion

In this work, we had successfully synthesized single crystalline Mg_3_Bi_2_ by Bi‐flux method, and carried out measurements of thermal and thermoelectric properties. It is shown that the resistivity of those samples have metallic behavior, with concentration of 1.88–3.16 × 10^20^ cm^−3^ and Hall mobility of 28.5–54.2 cm^2^ V^−1^ s^−1^ for carriers at room temperature. Above 100 K, polar‐optical phonons mainly scatter diffusive electrons, due to relationship of *μ*∼*T* ^−1^. For the most of samples, lattice thermal conductivity approaches to *κ_L_* ∼*T* ^−0.5^ above 100 K, deviated from phonon‐phonon interaction, and impurities scattering would be dominating mechanism for thermal conduction, probably attributed to high density of Mg vacancies. The lowest *κ_L_* is ≈0.49 W m^−1^ K^−1^ for S6 at 285 K, with highest carrier mobility of ≈54.2 cm^2^ V^−1^ s^−1^ around room temperature, and the *L*_ph_ is estimated to ≈0.48 nm, approaching to the minimum of atomic distance in crystal structure. In those samples, it is found that Seebeck coefficient has a “hump” ≈20 K, driven by the phonon drag effect. The contributions to *S* from phonon drag part are variable in samples, probably due to the difference of defects’ density. It is shown that *S*_ph_ is positively proportional to *L*_ph_, and the coefficient of *S*_ph_/*L*_ph_ is ≈4.6 × 10^2^ µV K^−1^ µm^−1^ in Mg_3_Bi_2_, twice that in FeSb_2_ (≈1.9 × 10^2^ µV K^−1^ µm^−1^) and CrSb_2_ (≈1.7 × 10^2^ µV K^−1^ µm^−1^), although the additional enhancement of *S* is quite low. The strength of electron‐phonon interaction in single crystalline Mg_3_Bi_2_ is estimated to ≈0.12. The *L*_ph_‐intercept of the linear fit to *S*_ph_ is ≈4.68 nm, less than the mean free path of electrons, where *S*_ph_ is negligible with phonons mostly interacting with boundaries and defects. It is believed that defects of Mg vacancies, introduced during materials’ synthesis process, mainly limit the mean free path of phonons, therefore lead to a trivial *S*_ph_ in single crystalline Mg_3_Bi_2_. Minimizing the grain size and reducing the density of Mg vacancies would give a longer *L*_ph_, therefore making that the phonon drag effect could drive a significant enhancement of Seebeck coefficient at low temperature in single crystalline Mg_3_Bi_2_. In the future, the synthesis strategy for optimizing the quality of single crystals is in need of further investigation on Mg_3_Bi_2_. The findings would pave a way on insight of fundamental thermoelectric transport in single crystalline metallic materials, for “phonon‐glass electron‐crystal” thermoelectric compounds with low lattice thermal conductivity and high carrier mobility.

## 4 Experimental Section

### Materials synthesis

Single crystalline Mg_3_Bi_2_ samples were grown using a self‐flux method with excess Bi as flux.^[^ ^22^, ^54^ ^]^ High‐purity magnesium shots (Mg, 99.8%, Alfa Aesar) and bismuth shots (Bi, 99.999%, Alfa Aesar) were weighed and mixed with different molar ratios of Mg and Bi (see Table SI, Supporting Information). The mixture was put into alumina or tantalum crucible in an argon glove box and then sealed in a silica ampoule under a partial pressure of argon. The ampoule was heated to 650 °C over 6 h, kept for 24 h, and then cooled slowly down to 350 °C at a rate of 1.5–2.5 °C h^−1^. Subsequently, after removing the Bi flux in a centrifugation process, several single crystalline flat flakes with a lateral size of ≈6–8 mm were obtained.

### Sample Characterization

The phase quality of the samples was checked by X‐ray diffraction (XRD) under Bruker D2 Advance X‐ray diffractometer with Cu‐K_𝛼_ radiation. Chemical compositions were analyzed by scanning electron microscope (SEM, Carl Zeiss) with an attached energy‐dispersive X‐ray spectrometer (EDS). The actual composition was determined by the inductively coupled plasma optical emission spectroscopy (ICP‐OES, Thermo). The electrical resistivity and carrier concentration were measured using the Physical Properties Measurement System (PPMS Dynacool, Quantum Design) with electrical transport option. Thermal conductivity and Seebeck coefficient were measured using PPMS with thermal transport option (TTO), after a careful correction for heat radiation loss. In the measurements, the estimated errors of thermal conductivity from radiative heat loss were less than 1% around room temperature. The concentration (*p*) and electronic Hall mobility (*μ*) of carriers are calculated by the formula of *p* = 1/*eR*_H_ and *μ* = 1/*ρpe*, respectively, where *e* is elementary charge, *R*_H_ is Hall coefficient, and *ρ* is electrical resistivity. Lattice thermal conductivity (*κ_L_*) was obtained by subtracting electronic thermal conductivity (*κ*_e_) from total thermal conductivity, where *κ*_e_ was calculated using Wiedemann–Franz law, *κ*_e_ = *L*_0_ *T/ρ*, with Lorentz number *L*_0_ estimated using an equation: $L_{0}=1.5+exp\left[-\frac{\left|S\right|}{116}\right]$, based on the experimental data of Seebeck coefficient, where *L*_0_ and *S* are in 10^−8^ W Ω K^−2^ and µV K^−1^, respectively. The mean free path of phonons (*L*_ph_) was calculated by the kinetic theory of phonons gas model, *κ_L_* = *CυL*_ph_/3, where *C* is specific heat capacity, and *υ* is group velocity of phonons, from experimental results in references.^[^ ^18^, ^55^ ^]^ All the samples were stored in a glovebox with both moisture and oxygen contents below 1 ppm, and the measurements were conducted under high vacuum conditions in PPMS equipment, which largely avoided the influences of instability in air.^[^ ^56^, ^57^ ^]^

## Author Contributions

H.L. conceived the project and designed the experiments. Q.F. and H.L. synthesized the single crystalline samples, performed thermal and thermoelectric measurements, and analyzed the data. H.L. and Q.F. wrote the manuscript. All authors contributed to discussing the data and editing the manuscript.

## Conflict of Interest

The authors declare no conflict of interest.

## Supporting information



Supporting Information

## Data Availability

The data that support the findings of this study are available from the corresponding author upon reasonable request.
