# Supplementary material for: Low Thermal Conductivity in Single Crystalline Mg3Bi2 and Its Thermopower Enhanced by Electron‐Phonon Interaction
Source: Adv Sci (Weinh). 2025 May 5;12(22):2416518. doi: 10.1002/advs.202416518 (PMC12165069; doi:10.1002/advs.202416518)
Supplement: Supplementary file 1 — Supporting Information [file ADVS-12-2416518-s001.pdf]

ADVANCED SCIENCE
Open Access

Supporting Information

for *Adv. Sci.*, DOI 10.1002/advs.202416518

Low Thermal Conductivity in Single Crystalline Mg_3Bi_2 and Its Thermopower Enhanced by Electron-Phonon Interaction

*Qiang Feng, Jiayi He, Wenyang Wang and Huili Liu**

Supporting Information (SI)

Low Thermal Conductivity in Single Crystalline Mg_3Bi_2 and Its Thermopower Enhanced by Electron-Phonon Interaction

Qiang Feng,[1] Jiayi He,[1] Wenyang Wang,[1] & Huili Liu[1,*]

[1]*School of Physical Science and Technology, ShanghaiTech University, Shanghai 201210, China*

[*]Author to whom correspondence and material requests should be addressed. E-mail: liuhl3@shanghaitech.edu.cn

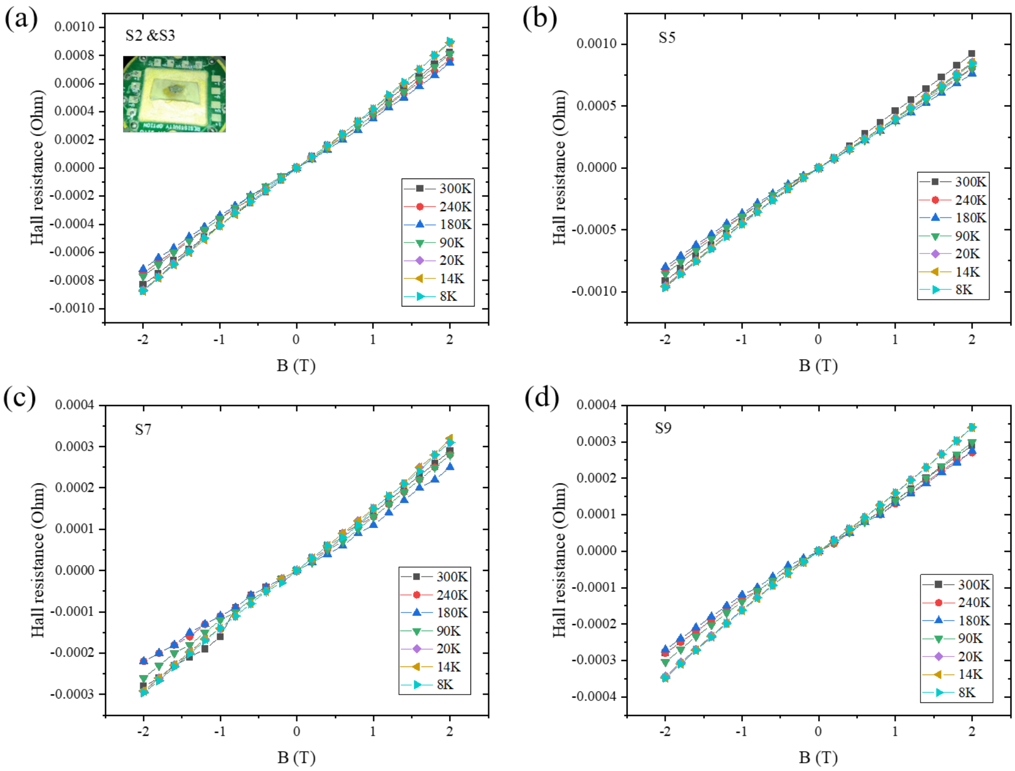

Figure S1. Hall resistances as a function of magnetic field for samples S2&S3, S5, S7 and S9 at low temperature. Inset in (a) is an optical image of Hall device for measurements.

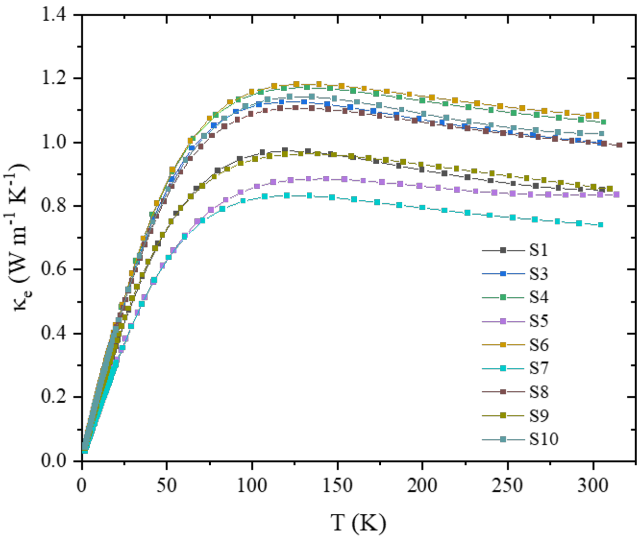

Figure S2. In-plane electrical thermal conductivity as a function of temperature in single crystalline Mg_3Bi_2.

Table SI. The synthesis profiles and results for samples S1-S10.

Sample	Crucible	Synthesis profile	Nominal Composition	Actual Composition (EDS)	Actual Composition (ICP)
S1	alumina	$\mathrm{RT} \rightarrow 650 \xrightarrow{24\mathrm{h}} 650 \xrightarrow{2.5^{\circ}\mathrm{C/h}} 350$	$Mg_{18}Bi_{82}$	$Mg_{2.84}Bi_2$	$Mg_{2.78}Bi_2$
S2	alumina	$\mathrm{RT} \rightarrow 650 \xrightarrow{24\mathrm{h}} 650 \xrightarrow{2.5^{\circ}\mathrm{C/h}} 350$	$Mg_{18}Bi_{82}$	$Mg_{2.89}Bi_2$	$Mg_{2.79}Bi_2$
S3	alumina	$\mathrm{RT} \rightarrow 650 \xrightarrow{24\mathrm{h}} 650 \xrightarrow{2.5^{\circ}\mathrm{C/h}} 350$	$Mg_{18}Bi_{82}$	$Mg_{2.89}Bi_2$	$Mg_{2.79}Bi_2$
S4	alumina	$\mathrm{RT} \rightarrow 650 \xrightarrow{24\mathrm{h}} 650 \xrightarrow{2.5^{\circ}\mathrm{C/h}} 350$	Mg_1Bi_3	$Mg_{2.80}Bi_2$	$Mg_{2.86}Bi_2$
S5	alumina	$\mathrm{RT} \rightarrow 650 \xrightarrow{24\mathrm{h}} 650 \xrightarrow{2.5^{\circ}\mathrm{C/h}} 350$	Mg_1Bi_2	$Mg_{2.82}Bi_2$	$Mg_{2.89}Bi_2$
S6	alumina	$\mathrm{RT} \rightarrow 650 \xrightarrow{24\mathrm{h}} 650 \xrightarrow{2.5^{\circ}\mathrm{C/h}} 350$	Mg_1Bi_3	$Mg_{2.80}Bi_2$	$Mg_{2.86}Bi_2$
S7	alumina	$\mathrm{RT} \rightarrow 650 \xrightarrow{24\mathrm{h}} 650 \xrightarrow{1.5^{\circ}\mathrm{C/h}} 350$	Mg_1Bi_2	$Mg_{2.86}Bi_2$	$Mg_{2.93}Bi_2$
S8	alumina	$\mathrm{RT} \rightarrow 650 \xrightarrow{24\mathrm{h}} 650 \xrightarrow{2.5^{\circ}\mathrm{C/h}} 350$	Mg_1Bi_4	$Mg_{2.78}Bi_2$	--
S9	tantalum	$\mathrm{RT} \rightarrow 650 \xrightarrow{24\mathrm{h}} 650 \xrightarrow{1.5^{\circ}\mathrm{C/h}} 350$	Mg_3Bi_7	$Mg_{2.70}Bi_2$	--
S10	alumina	$\mathrm{RT} \rightarrow 650 \xrightarrow{24\mathrm{h}} 650 \xrightarrow{1.5^{\circ}\mathrm{C/h}} 350$	Mg_1Bi_3	$Mg_{2.76}Bi_2$	--

Table SII. The parameters of the fit to experimental Seebeck coefficient for samples S1-S10 using Equations (1) – (4) in main text.

Sample	A (μV K^{-2})	B (μV K^{-4})	C (μV K^{-1})	D	E (μV $K^{-1.5}$)
S1	0.1366	8.57E-5	1.25	8.928E-5	0.0212
S2	0.0425	2.25E-4	1.23	1.617E-4	0.4884
S3	0.1227	6.83E-5	1.10	9.363E-5	0.0025
S4	0.1358	1.02E-4	1.40	8.709E-5	0.0227
S5	0.0931	8.50E-5	0.75	1.990E-4	-0.0143
S6	0.0969	1.32E-4	1.30	1.093E-4	0.1820
S7	0.1068	1.13E-4	1.80	5.216E-5	0.5226
S8	0.0840	1.22E-4	1.10	2.344E-4	0.1795
S9	0.1090	1.03E-4	1.10	1.886E-4	-0.0562
S10	0.1066	7.49E-4	0.96	2.143E-4	-0.0326
